# The Implementation of a Geriatrics Co-Management Model of Care Reduces Hospital Length of Stay

**DOI:** 10.3390/healthcare10112160

**Published:** 2022-10-29

**Authors:** Homero Teixeira Leite, Alex C. Manhães, Luisa A. Antunes, Tevy Chan, Guy Hajj-Boutros, José A. Morais

**Affiliations:** 1 Prevent Senior, Av. Jorge Curi, 550–Bloco A–Sala 186–Barra da Tijuca, Rio de Janeiro 22611-202, Brazil; 2 Department of Physiological Science, Universidade do Estado do Rio de Janeiro, Rio de Janeiro, Avenida Marechal Rondon, 381, São Francisco Xavier, Rio de Janeiro 0950-000, Brazil; 3 Division of Geriatric Medicine, McGill University, Montreal, QC H3G 1A4, Canada; 4 Research Institute of the McGill University Health Centre (MUHC), Montreal, QC H4A 3J1, Canada

**Keywords:** geriatrics co-management, inpatient, vulnerable, length of stay, hospital costs

## Abstract

(1) Background: Older adults comprise a large proportion of hospitalized patients. Many are frail and require complex care. Geriatrics has developed models of care specific to this inpatient population. Our objective was to demonstrate the effect of a geriatric co-management team on clinical administrative indicators of care in Clinical Teaching Units (CTUs) that have adopted the Age-friendly Hospital (AFH) principles in Brazilian hospitals. (2) Methods: Following 3 months of implementation of the AFH principles in CTUs, two periods of the same 6 months of two consecutive years were compared. (3) Results: The total number of participants in the study was 641 and 743 in 2015 and 2016, respectively. Average length of patient-stay (length of stay: 8.7 ± 2.7 vs. 5.4 ± 1.7 days) and number of monthly complaints (44.2 ± 6.5 vs. 13.5 ± 2.2) were significantly lower with the co-management model. Number of homecare service referrals/month was also significantly higher (2.5 ± 1 vs. 38.3 ± 6.3). The 30-day readmission rates and total hospital costs per patient remained unchanged. (4) Conclusion: The presence of a geriatric co-management team in CTUs is of added benefit to increase the efficiency of the AFH for vulnerable older inpatients with reduced LOS and increased referrals to homecare services without increasing hospital costs.

## 1. Introduction

The activity of present acute care hospitals reflects the changes occurring in our society in which treatments and services are being provided to patients of ever-greater age using advanced technology. The present model of hospital care often presents hostile characteristics to older patients [1]. What have been reported with greater frequency are, (a) immobilization contributing to rapid loss of muscle mass [2]; (b) the association of chronic diseases and potential geriatric syndromes such as delirium, dementia, postural instability and neurosensorial hearing impairment, that makes the functioning of the hospital unsuitable to this type of patients with complexity [2]. In response to this challenge, there is a tendency to promote overuse of a system with multiple specialists, unnecessary complementary tests and polypharmacy that may reflect the difficulty in maintaining focus in the treatment that brought the older patient to the hospital, in preventing iatrogenesis and in promoting a more safe and efficient stay; (c) the fragmented hospital style operating with several departments that results in a reduction in efficacy, amplification of costs and slowing in the communication between professionals [3]; (d) and lastly, the difficulty of healthcare professionals in maintaining a privileged listening to shared treatment decisions with older patients and their families aiming at more appropriate decisions [4,5].

Those above 75 years of age have in average 4–6 chronic diseases [6] and not so infrequently display geriatric syndromes that predispose them to loss of functional capacity. Brazil is a developing country whose population is aging at a fast pace and the characteristics of the hospitalized older patient are in many aspects similar to that of more developed countries. In many instances, following a hospitalization, the older adult is less likely to live in the community and exercise their autonomy and independence in full [6,7]. This situation has stimulated the improvement of the approach to the older adult at risk of functional decline by implementing models of care such as Acute care of elders (ACE) [8] geriatrics co-management and the Age-friendly Hospital (AFH) concept [9]. Geriatric co-management has been considered an alternative approach, and is defined as a shared responsibility and decision making between at least a treating physician (e.g., surgeon) and a geriatrician who provides complementary medical care in the prevention and management of geriatric-oriented problems [9].

Most studies on the geriatrics co-management model of care were done in the surgical setting, such as cancer-related surgeries, hip fractures and trauma. In these studies, geriatrics co-management was shown to be associated with improved postoperative outcomes including decreased postoperative complications, mortality, hospital length of stay (LOS) as well as readmissions [10]. However, very few studied the effect of geriatrics co-management model in the medical setting. 

Internal medicine Clinical Teaching Units (CTUs) receive many older patients with multiple chronic conditions and geriatric syndromes, and would be an ideal setting to apply the AFH principles and geriatrics co-management model. In CTUs of the Hospital Adventista Silvestre, Rio de Janeiro, Brazil, AFH principles have been implemented in 2013. Our objective was to demonstrate the effect of geriatric co-management on clinical administrative end-points in the internal medicine CTUs that have already adopted the AFH principles. This is a quality improvement study assessing pre and post co-management impact on several endpoints. These end-points were hospital LOS, readmission rate at 30 days, home care services utilization upon discharge, recourse to Court orders by families to obtain home care services, use of the hospital ombudsman office for conflicts perceived by patients and their families and cost–benefit per hospital stay. Some of these end-points were related to answer specific needs of the hospital. 

## 2. Materials and Methods

### 2.1. Age-Friendly Hospital Principles Implantation in CTUs of Hospital Adventista Silvestre

Hospital Adventista Silvestre is a private, acute care hospital of 141 beds whose administration also runs a geriatric ambulatory and homecare service called Unidade Integrada de Prevenção (UIP). In 2013, efforts were made to link UIP with the internal medicine CTUs of the hospital, in order to ensure better post-discharge services. The CTUs are responsible for 70 inpatient beds, of whom 90% are occupied by patients older than 70 years. 

Later in the same year, one of the authors (HTL) helped the CTUs to adopt AFH principles after receiving training in the Division of Geriatric Medicine of McGill University, Montreal, Canada. The following year, 2014, can be considered a transition period before implementation of AFH where several training sessions were given by HTL to the CTU treating teams and adjustments of processes took place, such as encouraging patient mobility. 

### 2.2. The Geriatrics Co-Management Intervention

From January 2015 to June 2016, the geriatric consultation team, composed of a certified geriatrician (HTL), clinical nurse specialist, physiotherapists and geriatrics fellows proceeded with screening for vulnerability of all CTUs inpatients aged > 60 years. Assessments were based on the PRISMA 7 instrument [11], and those with a score > 3 were considered vulnerable. During the first period of 1 January to 30 September 2015, the interventions were mainly to guide the CTU treating team in implementing the AFH principles and served as baseline data to which to compare that of the second and more active period of intervention from 1 October to 30 June 2016. During this second period, the care was co-managed with the treating team as described under the roles of each geriatric team members with a focus on those vulnerable patients with prolonged hospital stay > 20 days.

Several geriatric tools were employed to improve monitoring and provide a geriatric vision while gathering information on the CTUs. These included the Katz activities of daily living [12] for assessing capacities on the last 30 days prior to admission, a recognized test validated against nurses’ judgment [13]. The Mini-Nutrition Assessment (MNA)–short form [14] for defining the nutritional status which has sensitivity of 98% and specificity 100%, respectively [15]. In addition, the Confusion Assessment Method for delirium, which has a sensitivity of 94% and specificity 89%, respectively [16]. A standard Hospital-UIP file was created for each patient to exchange and integrate information between these different settings of care. 

### 2.3. The Role of the Geriatric Team

The co-management was chaired by HTL who was the project leader of the implementation of the AFH program and coordinator of the activities between the Hospital and the UIP. From 1 October 2015 onward his role consisted of (1) assist the treating team on the interdisciplinary therapeutic plan focusing on medication reconciliation, maintenance of functional capacity of older adults, discharge plan and adequacy of home care support post-discharge; (2) organize continuing education activities for all health professionals of the hospital with emphasis on the AFH and other gerontological topics related to the specific needs of the clinical cases or the CTU team; (3) orient and supervise exchange residents rotating in geriatrics; (4) hold monthly meetings with UIP for cases with home care support after discharge to better define conduct and long-term plan for the most complex cases; and (5) stimulate the whole team of students, residents and staffs to consider the gerontological-geriatrics approach in the multidisciplinary rounds that occur 3 times a week [17,18].

### 2.4. Data Collection

For comparison purposes, we selected 6 months of data collection for each of the two intervention periods after allowing at least 3 months for learning and adaptation of processes and aiming at the same months of the year. Thus, baseline data collection (Period 1) went from 1 January to 30 June 2015 and the effect of the geriatric consultation team (Period 2) from 1 January to 30 June 2016. The total number of occupancies in patient days per month, the number of home care support/month and the total number of complaints per month in addition to other parameters related to the intervention were collected during the two years intervention. Data was collected on the clinical administrative end-points by the geriatric clinical nurse specialist on daily basis and entered into a spreadsheet to be later analyzed. Cost–benefit data were supplied by the administration office of the hospital that keeps detailed records of every item and intervention during hospitalization. The study received approval from the director of professional service as it was considered a quality improvement project. 

### 2.5. Statistical Analyses

Data are presented as mean with standard deviations. Normality of data distribution was assured by the Kolmogorov–Smirnov test. All data followed a normal distribution. Independent *t*-tests and chi-square were used to assess differences between the two index periods of data collection for each of the endpoints. The main comparison using the independent t-tests was to analyze the differences between the consecutive years in the total number of patient days occupancy, the average length of stay, the number of post discharges legal cases and the number of home care support. Analyses were done using SPSS 25.0 for Windows (SPSS, Chicago, IL, USA). Significance was set at *p* < 0.05.

## 3. Results

**Figure 1:**
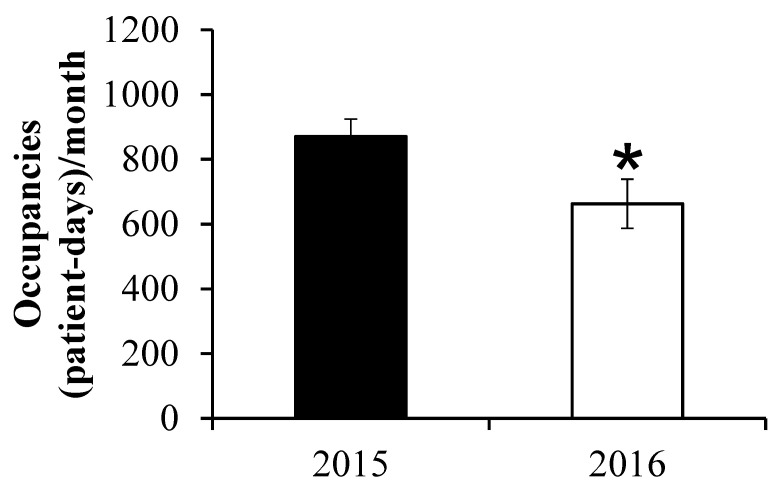
Total number of occupancies in patient days per month. Reference: * *p* < 0.05.

**Figure 2:**
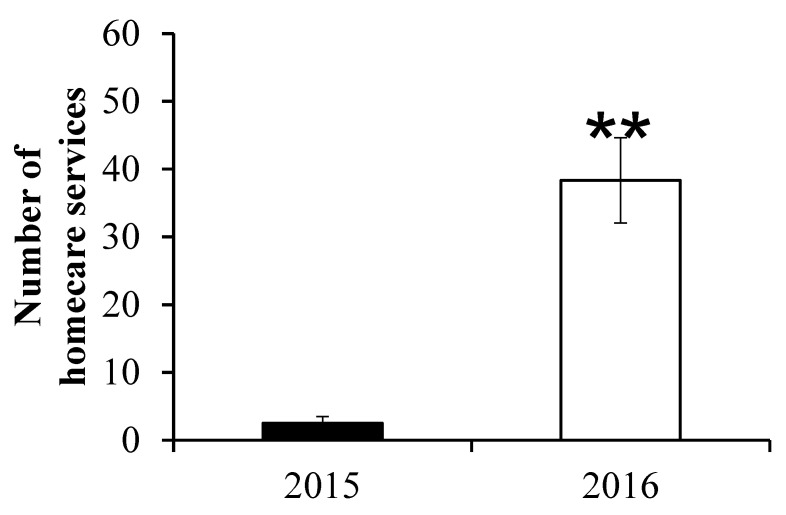
Number of home care support/month. ** *p* < 0.01.

**Figure 3:**
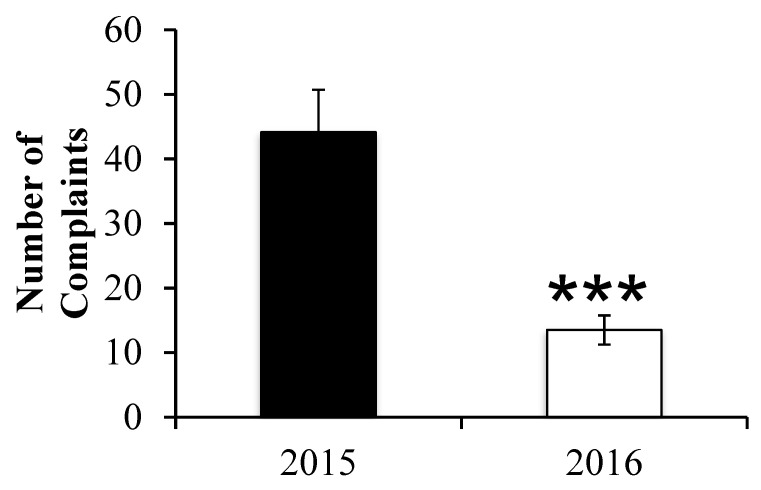
Total number of complaints per month. *** *p* < 0.001.

The clinical administrative data are presented in Table 1. All patients admitted to the hospital were included in the analysis. A total of 641 and 743 patient were admitted for the 2015 and 2016 periods, respectively. We did observe a significant reduction in 2016 compared to 2015 in the total number of patient days occupancy per month (662.8 ± 185.2 vs. 870.3 ± 132.2; *p* = 0.04; see also Figure 1) and the average length of patient-stay (5.4 ± 1.7 vs. 8.7 ± 2.7; *p* = 0.03). Both groups were comparable for the average number of patients admitted per month (*p* = 0.36), the percentage of readmission per month (*p* = 0.13) and the total hospital cost per patient (*p* = 0.20). The number of post-discharge legal cases decreased from 2015 to 2016 but numbers are too small to conduct meaningful analysis. Figure 2 discloses a significant increase in the number of home care support services in 2016 compared to 2015 (38.3 ± 6.3 vs. 2.5± 1.0; *p* < 0.01). Figure 3 depicts the reduction in the number of complaints from 2015 to 2016 (44.2 ± 6.5 vs. 13.5 ± 2.2; *p* < 0.001).

## 4. Discussion

The aim of this study was to determine if the presence of a multidisciplinary geriatric consultation team dedicated to the care of frail older inpatients in co-management with an Internal Medicine treating team was of added benefit on several clinical administrative end-points in a CTU that had adopted the AFH principles. We found that its presence did not only reduced average length of stay, therefore increasing patient flow, but also decreased the number of complaints by patients and their family and this, without incurring extra costs. Our results are in agreement with other studies, mainly in the surgical setting, disclosing that geriatrics co-management was associated with reducing LOS [9]. Such results were likely obtained by providing a more patient-centered care and implementing successful discharge planning as evidenced by the increased number of homecare services per month (Figure 2). Further, one of the AFH tenets rests on providing care that involves patients on the decision-making process [17], which should lead to greater satisfaction and therefore reduced complaints to the hospital ombudsman as observed. 

As the AFH was already being implemented, it is likely that the approach of the AFH concept when applied by experts in the field of geriatrics using the comprehensive geriatric assessment [17] contributed to bringing it to its full development. Basic approaches of care promoted by geriatrics such as early mobilization, emphasis on maintaining adequate nutrition and hydration as well as performing medication reviews and interacting with patients on a more frequent and humane way could have contribute to this success. Another way through which these positive results could have been achieved would be by reducing hospital-related complications, therefore making early discharge possible and this, independently from the availability of homecare resources. We did not assess any incident complications, such as delirium, falls or adverse medications events, but the purpose of the AFH is to maintain functional capacity that implicitly includes prevention of complications. Hospitalization in frail older adults is recognized for causing new disability in basic and instrumental activities of daily living [19] which are associated with adverse health events and risk of rehospitalization [20].

Several meta-analyses have proven the efficiency of the geriatric wards for frail inpatients by showing reduced rates of death at 6 months, functional decline, institutionalization and cognitive losses compared to usual care [21,22,23]. Less clear remains the usefulness of a geriatric mobile team model often composed solely of a geriatrician and a nurse [23]. In the present geriatric intervention however, it is a co-management model closer to the Acute Care of Elders (ACE) model since there was a geriatrician with his team assisting an internist and the treating team in patient care [8]. Similar to our findings, a clinical overview of the ACE units has shown to improved patient functional status at time of hospital discharge and reductions in length of stay in patients admitted to these units as compared to usual care [8] 

Our 30-day readmission rate was not significantly affected by the intervention despite a favorable trend (Table 1). It remains unclear if one was powered enough for this end-point as it is a significant marker of the quality of the discharge plan and homecare resources [24]. However, in the present study, rates of readmission are considered very good since only 9.2% and 4.6% were observed for the years 2015 and 2016, respectively, as opposed to 16.8% in one Canadian study [25] Other than the fact that it is always more difficult to find differences when rates are already low at baseline, it also reflects the benefit of having an integrated care system to prevent readmissions [26]. However, these results are in contrast with those of an ACE unit data showing significant decreased 30-d readmission rates [27].

Of interest is that despite the increased presence of healthcare staff on the CTUs (geriatrician, nurse and rehabilitation professionals) whose cost was factored in the expense analysis, the intervention remained cost neutral based on total costs for the duration of hospitalization or by individual patient (Table 1). In contrast to our neutral cost, one study showed that ACE unit is cost-effective [8]. Further, the costs of the homecare services were not accounted for, which would have contributed to elevate the cost of the intervention. Regardless, it is reassuring that despite requiring increased human resources, the effectiveness of the co-management intervention in producing early discharges from the hospital without increasing readmissions were not associated with greater expenses. Results of this study supports the notion that on hospital wards with many older inpatients having a geriatric team involved in a co-management care model is an asset to improve efficacy of the care.

This study has several strengths that worth mention, in particular, its prospective design, the reliability of the hospital data acquisition entered on daily basis and the careful evaluation of the costs by the hospital administration. Among its limitations, one would need to be cognizant of the non-randomized nature of the study design and of the context of a private hospital well-integrated in a healthcare network system that would require further replication of the findings in a different context before concluding in its generalization.

## 5. Conclusions

Overall, we demonstrated the usefulness of having a model of co-management with geriatrics in the implementation of the AFH concept in a CTU with many older inpatients. Results bring evidence to include geriatric co-management care model in wards with many older patients as it decreases average length of stay and patient’s complaints to the ombudsman without increasing 30-d readmissions and this, while being cost-neutral. 

## Figures and Tables

**Table 1:** Clinical administrative data.

Variables	2015	2016	*p* Value
Total number of patients admitted (n)	641	743	-
Average number of patients admitted/month	106.8 ± 24.8	123.8 ± 21.8	0.36
Total number of patient days occupancy/month	870.3 ± 132.2	662.8 ± 185.2	**0.04**
Average length of patient-stay (days)	8.7 ± 2.7	5.4 ± 1.7	**0.03**
Readmission at 30 days (n)	36	26	-
30-day readmission/month	6.0 ± 3.1	4.3 ± 1.0	0.33
% Readmission/month	9.2 ± 6.8	4.6 ± 1.1	0.13
Post-discharge legal cases	4	1	-
Total hospital costs (USD)	2,054,140 ± 83,620	1,820,350 ± 119,310	0.52
Total hospital costs/patient (USD)	3018 ± 1363	2319 ± 1114	0.20

Mean numbers and ± standard deviation is presented in the table.

## Data Availability

Data were archived at Unidade Integrada de Prevenção, Rio de Janeiro, Brazil.
